# Genetically predicted triglycerides mediate the relationship between type 2 diabetes Mellitus and intervertebral disc degeneration

**DOI:** 10.1186/s12944-023-01963-4

**Published:** 2023-11-14

**Authors:** Ding-Qiang Chen, Wen-Bin Xu, Xin Chen, Ke-Yi Xiao, Zhi-Qiang Que, Nai-Kun Sun, Jin-Yi Feng, Gang Rui

**Affiliations:** 1 The School of Clinical Medicine, Fujian Medical University, Fuzhou, China; 2 Department of Orthopedics, The First Affiliated Hospital of Xiamen University, School of Medicine, Xiamen University, Xiamen, China; 3 Department of Cardiac Surgery, The First Affiliated Hospital of Xiamen University, School of Medicine, Xiamen University, Xiamen, China

**Keywords:** Type 2 Diabetes Mellitus, Mendelian randomization analyses, Triglycerides, Intervertebral disc degeneration, Causality

## Abstract

**Background:**

To validate the causal relationship between type 2 diabetes mellitus (T2DM) and intervertebral disc degeneration (IVDD) and to identify and quantify the role of triglycerides (TGs) as potential mediators.

**Methods:**

A two-sample Mendelian randomization (MR) analyses of T2DM (61,714 cases and 1178 controls) and IVDD (20,001 cases and 164,682 controls) was performed using genome-wide association studies (GWAS). Moreover, two-step MR was employed to quantify the proportionate impact of TG-mediated T2DM on IVDD.

**Results:**

MR analysis showed that T2DM increased IVDD risk (OR: 1.0466, 95% CI 1.0049–1.0899, *P* = 0.0278). Reverse MR analyses demonstrated that IVDD does not affect T2DM risk (*P* = 0.1393). The proportion of T2DM mediated through TG was 11.4% (95% CI 5.5%-17.4%).

**Conclusion:**

This work further validates the causality between T2DM and IVDD, with a part of the effect mediated by TG, but the greatest impacts of T2DM on IVDD remain unknown. Further studies are needed to identify other potential mediators.

**Supplementary Information:**

The online version contains supplementary material available at 10.1186/s12944-023-01963-4.

## Introduction

Owing to the aging of the population, the number of people suffering from IVDD is growing dramatically. The major characteristic of IVDD is chronic low back pain (LBP), which greatly impacts patients’ daily lives and poses a great challenge to public health and socioeconomics. Despite the intricacy and diversity of the etiology of spine-related disorders, IVDD has been considered to be one of the top etiologic factors [1]. IVDD is a prevalent degenerative disease featuring a gradual reduction in protein glycans and moisture content in the nucleus pulposus [2] and consequent rupture of the discs between the vertebrae, inducing pressure on spinal nerves. The present conservation therapies or operative interventions are unable to reverse IVDD [3].

DM is a systemic disease caused by defective insulin secretion or insulin resistance, and approximately 90% of diabetes patients have type 2 diabetes [4, 5]. DM is a multiorgan disorder impacting multiple tissues and organs, including bone and cartilage [6]. A controlled study by Liu and his team involving 772 participants, as well as a retrospective-study in India found that a duration of T2DM of > a decade and poorly controlled T2DM can increase IVDD risk. In addition, a 4-year longitudinal study from the Wakayama Spine Study further confirmed the strong association between DM and the incidence of IVDD [7–9]. IVDD is a serious medical issue [10] that often causes moderate to severe pain in patients. This pain not only affects their quality of life but also increases the corresponding medical costs [11, 12]. However, traditional approaches have focused on treating IVDD based on the symptoms [13]. Accordingly, it is particularly critical to identify risk factors for IVDD and understand the association between them and IVDD to prevent or delay the occurrence or development of IVDD. This could reduce the prevalence of IVDD and minimize the burdens on the health care system and socioeconomic system. Therefore, it is of great practical importance to study and validate the association between T2DM and IVDD as early as possible. In addition, several retrospective studies based on large populations have shown that TG impacts the degradation level and increases the probability of lumbar disc herniation in patients with IVDD [14, 15]. There is also a strong link between elevated TG levels and DM [16, 17]. Thus, TG may be a potential mediator between T2DM and IVDD. However, traditional epidemiologic studies are susceptible to instrument inaccuracies, uncontrollable confounders, and inverse causation, which eventually cause instability of the results. Research regarding the effect of lipid factors in mediating the association between T2DM and IVDD is also scarce, and thus, studies designed to avoid bias are needed to confirm the association between T2DM and IVDD as well as the mediating factors involved.

MR is a novel research method. It utilizes genetic variants linked to target traits to assess the causality between exposures and outcomes as instrumental variables (IVs). Compared with traditional observational studies, MR is extensively utilized in investigating potential causality between exposure and outcome because of its higher confidence in inferring causality, overcoming confounders, measurement error, and reverse causality issues [18]. The two-sample MR approach requires separate extraction of genetic variation in exposure and outcome in diverse datasets, which enables it to verify the causality between T2DM and IVDD with more robust statistical efficacy. In addition, the proportion of the impact of T2DM on IVDD that may be mediated through TG was quantified, which better identifies the role of triglycerides in this pathway.

## Materials and methods

### Study design

The research utilized the Twosample TwosampleMR [19] software package, an R language program package designed to estimate the causality between exposures and outcomes through the use of GWAS generalization data. Single nucleotide polymorphisms (SNPs) are genetic tools for inferring exposure impacts on outcomes.

### Data sources and selection of SNPs

The GWAS pooled data relevant to the purposeful characterization of the European population were obtained from open databases. GWAS databases with a larger sample size and number of SNPs were prioritized. T2DM-related data (Xue A et al., 2018) came from a European Bioinformatics Institute database meta-analysis involving three large T2DM GWASs with a total of 655,666 individuals [20]. TG-relevant data (Richardson et al., 2020) were obtained from the GWAS of the characterization of nonfasting cyclic lipoprotein lipids via the UK BioDatabase, involving more than 441,016 subjects [21]. The IVDD data came from the FinnGen database and involved 184,683 individuals, including 20,001 patients with IVDD and 164,682 controls (Table 1). First, SNPs matching the whole genome prominence threshold (*P* < 5 × 10^− 8^) requirement were included. To prevent the influence of strong linkage disequilibrium (LD), an LD threshold (r^2^ < 0.001) was set. In addition, SNPs whose F-statistic was > 10 were utilized to prevent weak instrumental bias [22] (Table S1-4).

**Table 1:** Summary of GWAS datasets included in this study

Phenotype	Sample size (case/control)	Number of SNPs	Population	Units	Databases
Exposure					
Type 2 diabetes (ebi-a-GCST006867)	655,666 (61,714/1,178)	5,030,727	European	logOR	EBI database
Mediation					
Triglycerides (ieu-b-111)	441,016	12,321,875	European	Not applicable	UKB database
Outcome					
Intervertebral disc degeneration (finn-b-M13_INTERVERTEB)	184,683 (20,001/164,682)	16,380,337	European	Not applicable	FinnGen database

SNP, single nucleotide polymorphism; Name of data set: ebi-a-GCST006867, ieu-b-111, finn-b-M13_INTERVERTEB

### Primary analysis

**Fig. 1:**
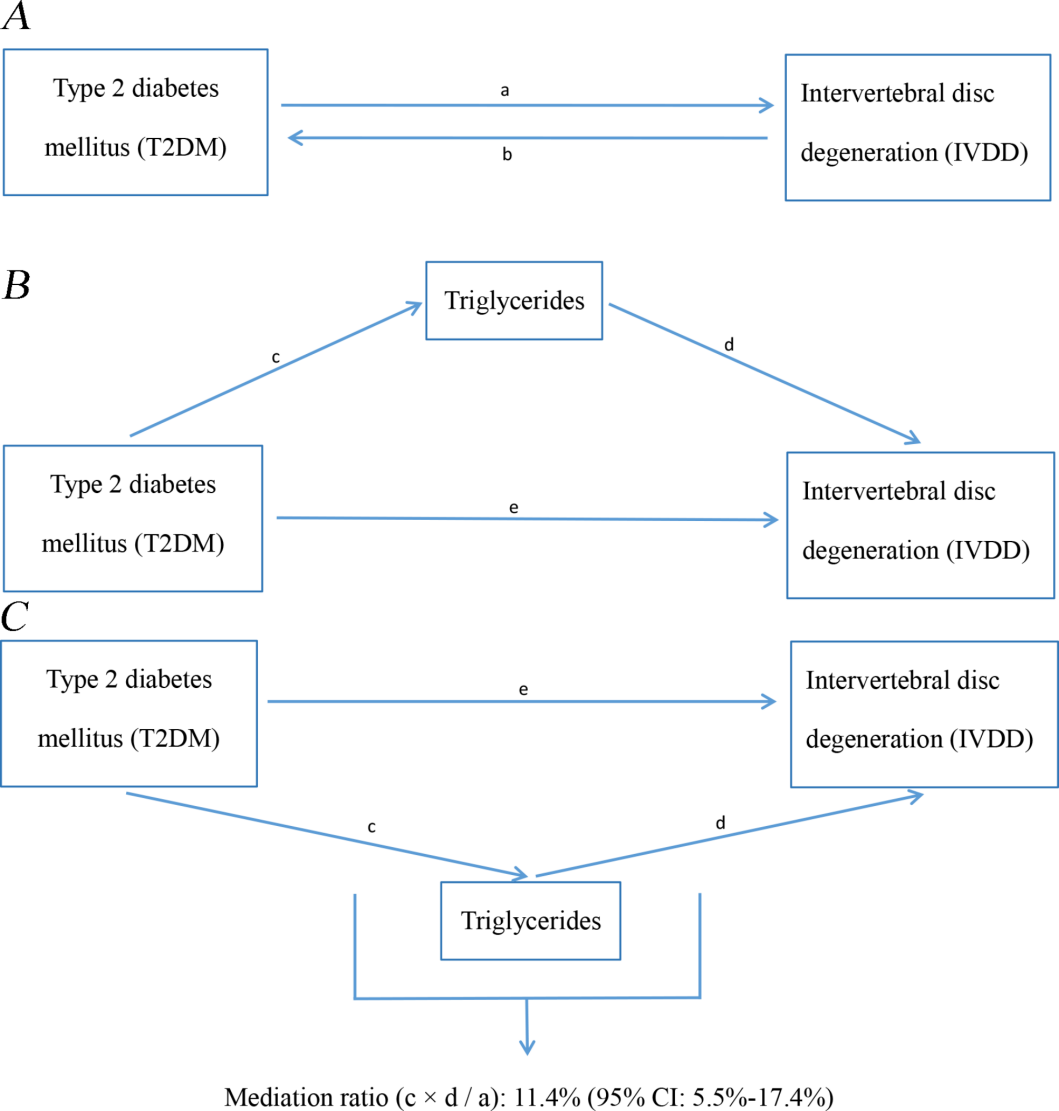
Diagram of analytical relationships. **(A)** The total influence between T2DM and IVDD. ‘a’ indicates the total influence of T2DM on IVDD. ‘b’ indicates the total influence of IVDD on T2DM. **(B)** Diagram of direct and indirect impacts. ‘c’ indicates the effect of T2DM on TG. ‘d’ indicates the effect of TG on IVDD (indirect impact = c × d). ‘e’ indicates direct effects = a - c × d. Mediation ratio = c × d / a

Two-sample bidirectional MR was utilized to evaluate the causality between T2DM and IVDD (Fig. 1A). The Inverse variance weighting (IVW) results were taken as the major observation. Furthermore, the MR‒Egger, weighted median, simple mode, and weighted mode approaches were used for verification.

### Mediation analysis

Mediation analysis was performed with two-step MR to test whether TGs were a mediator of T2DM on IVDD (Fig. 1B). The overall influence of T2DM on IVDD can be categorized into a direct impact of T2DM on IVDD (e in Fig. 1B) and an indirect (mediated) impact of T2DM through the mediator. The indirect effect was calculated as follows: the first step was to compute the impact of exposure on the mediator variable to obtain c, and the second step was to compute the impact of the mediator on the outcome to obtain d. The two coefficients then were multiplied together, yielding the indirect impact (c × d in Fig. 1B). The percentage mediated impact was then computed by dividing the indirect impact by the overall impact (c × d / a in Fig. 1C). A higher percentage of mediated effect indicates a larger proportion of the effect of T2DM affecting IVDD through TG. A 95% confidence interval was also calculated using the delta method [23].

### Sensitivity analysis

The IVW method has high reliability based on a combination of Wald estimations for every SNP via a method of meta-analysis, which yields a total estimation of exposure impact on the outcome [24]. However, the MR‒Egger approach, which is based on the Cochran’s Q statistics, primarily accounts for the dosage association of IVs with the outcome and considers partial pleiotropy [25]. Thus, heterogeneity was detected with the MR‒Egger and IVW approaches. Moreover, an enormous advantage of the MR‒Egger approach is that the presence of the intercept term is taken into account in the regression; thus, the MR Egger regression equation was utilized for evaluating the horizontal pleiotropy of the genetic tools. *P* > 0.05 indicated no heterogeneity and horizontal pleiotropy. Furthermore, MR‒pleiotropy residual sum outlier (MR‒PRESSO) testing was executed, which has high accuracy in identifying horizontal pleiotropy and outliers, and can reduce heterogeneity in causality estimates via exclusion of SNPs that cause heterogeneity [26]. Finally, to ensure that the stability of the results remained unaffected by individual SNPs, “leave-one-out” sensitivity analysis was conducted by removing each SNP individually and determining whether the results were stable.

### Statistical analysis

All analyses were completed in R 4.3.1. In addition, PhenoScanner [27] was employed for assessing all genetic tool-related phenotypes. The “Forestploter” package was used for forest mapping. The “MRPRESSO” package was used to detect outliers.

## Results

### Association between T2DM and IVDD

**Fig. 2:**
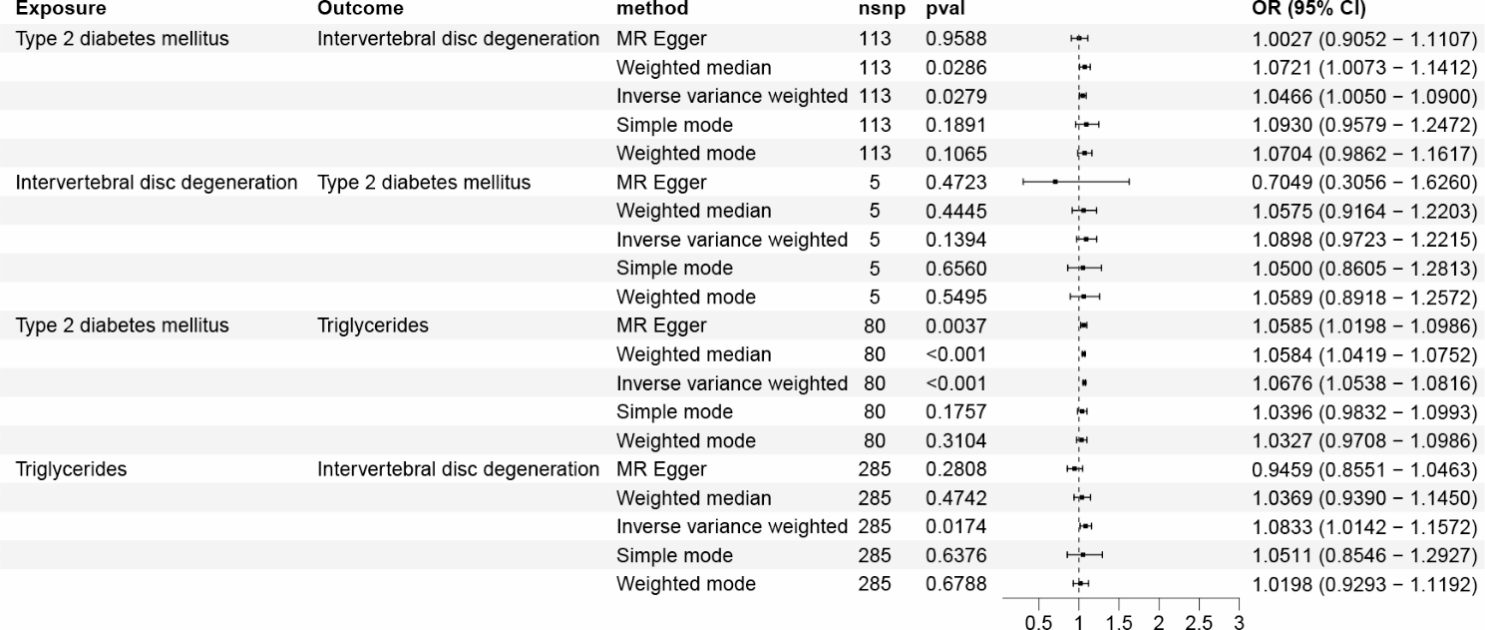
Forest plot of MR analysis results

The IVW approach results suggest that T2DM significantly increased IVDD risk (OR: 1.0466, 95% CI 1.0049–1.0899, *P* = 0.0278) and reverse MR analysis indicated no reverse causality for IVDD on T2DM (*P* = 0.1393). In addition, the remaining four MR analysis methods yielded similar results (Fig. 2 and Table S5).

### Association between T2DM and TG

The IVW approach results suggested an apparent causality between T2DM and TG (OR: 1.0676, 95% CI 1.0537–1.0816, *P* = 9.0024 × 10^− 23^). Furthermore, the remaining four MR analysis methods yielded similar results (Fig. 2 and Table S5).

### Association between TG and IVDD

TG was significantly related to IVDD (OR: 1.0833, 95% CI 1.0141–1.1571, *P* = 0.0174) with the IVW approach. The remaining four MR analysis methods yielded similar results (Fig. 2 and Table S5).

### The ratio of TG-mediated T2DM to IVDD association

The mediating role of TG in the correlation between T2DM and IVDD was analyzed. The analysis suggested that T2DM was related to elevated TG levels, which in turn were linked to an elevated IVDD risk. The calculations showed that TG accounted for 11.4% (95% CI 5.5%-17.4%) of the elevated IVDD risk associated with T2DM (Fig. 1C).

### Sensitivity analysis

Sensitivity analysis showed a degree of heterogeneity in some of the MR analysis processes (Table S6). The heterogeneity may arise from independent MR patterns of variation [28]. For example, when genes appear on the same chromosome, they can be linked to each other without following the law of independent assortment of heredity. In addition, IVs from different analysis platforms, experiments, populations, etc., may be heterogeneous and thus affect the results of Mendelian randomization analysis. The pleiotropy analysis shows weak pleiotropy evidence at the level of the orientation of the TG instrument. This suggests that some of the IVs may influence the outcome through other factors; however, the current technology is not yet able to fully explore the specific features involved in all SNPs. In addition, all the MR analysis procedures in this study were tested by MR-PRESSO analysis, and all SNPs that could lead to pleiotropy and heterogeneity were eliminated. Thus, the results of the present study still have strong credibility (Table S6). The leave-one-out test also demonstrated that the results were reliable (Fig. S1).

## Discussion

Recently several controlled surveys have been performed to examine the link between T2DM and IVDD. However, current studies are confined to the causality between the two, and there is uncertainty regarding the mediators between the two and the potential mechanisms. Therefore, within the current research, in addition to further validating the causality between T2DM and IVDD, two-step MR analysis was executed to analyze whether the causality between them is mediated through TG. The study results provide further evidence that T2DM is related to increased IVDD risk and suggest that 11.4% of this effect is mediated through TG.

LBP represents a worldwide problem that poses a serious health and socioeconomic challenge. Approximately 80% of the population develops this disease throughout their life. IVDD is currently recognized as the main cause of these symptoms [29]. Although mechanical compression, aging, genetics, autoimmunity, and toxicants have been demonstrated to increase IVDD risk, the link between systemic diseases and the pathogenesis of IVDD still requires further study [30–32]. Several prior observational investigations revealed a link between T2DM and IVDD [7–9]. The underlying mechanisms may be related to direct damage caused by reactive oxygen species (ROS), accumulated advanced glycosylation end products (AGEs), apoptosis, senescence, extracellular matrix (ECM) changes, hyperglycemia, obesity, and microvascular damage [33–35]. Zheng and his team found that the pathogenesis of IVDD is closely related to ROS by quantitating the ROS levels in human degenerated intervertebral discs via staining [36]. ROS formation by metabolizing oxygen in oxygen-utilizing cells is an inherent part of aerobic organisms, and ROS regulate homeostasis via multiple pathways, such as nuclear factor-κB, PI3K/AKT and protein kinase activation pathways [37]. Oxidative stress is an important element that induces procedural apoptosis, and its two main modes of death include apoptosis and autophagy, both of which play important roles in IVDD [38]. In addition, Huma Rizwan et al. found that high glucose levels could regulate mitochondrial dysfunction and apoptosis by enhancing ROS production [39]. In summary, T2DM may trigger the mitochondrial apoptotic pathway by increasing the level of ROS and thus induce apoptosis in nucleus pulposus and cartilage endplate (CEP) cells, ultimately resulting in the worsening of IVDD.

Dyslipidemia is a familiar characteristic of DM. In patients with diabetes, a correlation exists between atherosclerotic disorders and TG levels. Patients with diabetes are more susceptible to cardiovascular disease and hypertriglyceridemia at any given serum cholesterol level [40]. Thus, DM may exert subsequent effects by altering TG levels. In addition to the association with cardiovascular disease, there is a significant association between TG and degenerative IVD disease. A large observational study in Finland uncovered a link between elevated TG levels and sciatica [41]. In addition, a sizeable British cohort study revealed a marked correlation between TG and LBP, after adjusting for a variety of factors [42]. A follow-up report noted that TG could be a predictor of the morbidity of radiolucent LBP [43]. In addition, a case-control analysis demonstrated that lumbar disc herniation patients exhibited higher serum TG levels [44].

However, the detailed mechanism of the link between TG and IVDD is still unknown. Underlying links between IVDD and atherosclerosis due to dyslipidemia were discovered in prior research. Kauppila and his team found that atherosclerosis of the abdominal aorta, particularly segmental arterial orifice stenosis, may act as a causative agent for IVDD [45]. Additionally, a 25-year tracking report states that in the abdominal aortic posterior wall, calcified atherosclerotic sediments may increase IVDD risk and are related to LBP [46]. In addition, from an anatomical point of view, the lumbar vertebral body is supported by a branch of the lumbar artery. This artery stems from the bottom of the abdominal aorta. However, atherosclerosis tends to appear earliest in the lowest branches. As a result, atherosclerosis often occurs at or near the branch openings of the lumbar arteries, leading to lumbar artery segmental stenosis or occlusion [46, 47].

In summary, TG may be a mediator between T2DM and IVDD. Thus, a logical link between them may be that T2DM promotes atherosclerosis by increasing TG levels, which results in decreased blood availability to the relevant lumbar ganglia. This leads to IVD malnutrition and inadequate nutritional sustenance to IVD cells, eventually resulting in IVDD.

### Study strengths and limitations

This study has several strengths. The causality between T2DM and IVDD and the mediating role of TG were validated by MR analysis, and confounding by confounders and reverse causality was excluded. Exposure, outcome, and mediation data were obtained from GWAS research with large samples and SNP counts, guaranteeing the representativeness of IVs in the MR analysis. In addition, bias was minimized via a two-sample study design with no overlap of data at the exposures and outcomes. However, there are some unavoidable limitations to this study. First, the MR analyses used here were based only on online public database pooling data from studies with different analytical platforms, experiments, and populations from different regions of Europe, and thus, some heterogeneity is inevitable. Thus, the conclusions must also be tested in a large number of clinical trials. Second, the data were mainly based on European populations, and no subgroup analyses were performed on sex-specific populations. The SNPs extracted from the European population are not well representative of the populations of other continents due to differences in economic conditions, climate levels, and geography and large sex differences, which makes the application of this study to non-European and sex-specific populations somewhat limited. Finally, this study was mainly analyzed from the perspective of genetics, and the specific intrinsic mechanisms need to be explored in depth through more basic experiments in the future.

## Conclusion

In summary, this analysis provides further validation of the causality between T2DM and IVDD, where some of the impact is mediated by TG. While some mediation through TG is evident, a significant portion of the causal impact of T2DM on IVDD remains to be understood. Thus, further research is needed to elucidate other potential mediating elements. For the management of IVDD patients in clinical practice, while controlling blood glucose levels, proper attention should also be paid to TG levels. This will promote a better delay in the progression of IVDD and improve the overall prognosis and patients’ daily lives. Moreover, research on the interactions between T2DM, TG, and IVDD is still in its infancy, and more detailed mechanisms remain to be explored to further fill the gaps in the areas of prevention and delay of IVDD.

### Electronic supplementary material

Below is the link to the electronic supplementary material.

Supplementary Material 1

Supplementary Material 2

Supplementary Material 3

Supplementary Material 4

Supplementary Material 5

Supplementary Material 6

Supplementary Material 7

Supplementary Material 8

Supplementary Material 9

## Data Availability

Study data are accessible upon request from the corresponding author.

## Data Availability

All data are available from accessible online databases.
