# Supplementary material for: Genetically predicted triglycerides mediate the relationship between type 2 diabetes Mellitus and intervertebral disc degeneration
Source: Lipids Health Dis. 2023 Nov 14;22:195. doi: 10.1186/s12944-023-01963-4 (PMC10644578; doi:10.1186/s12944-023-01963-4)
Supplement: Supplementary file 7 — Supplementary Material 7 [file 12944_2023_1963_MOESM7_ESM.docx]

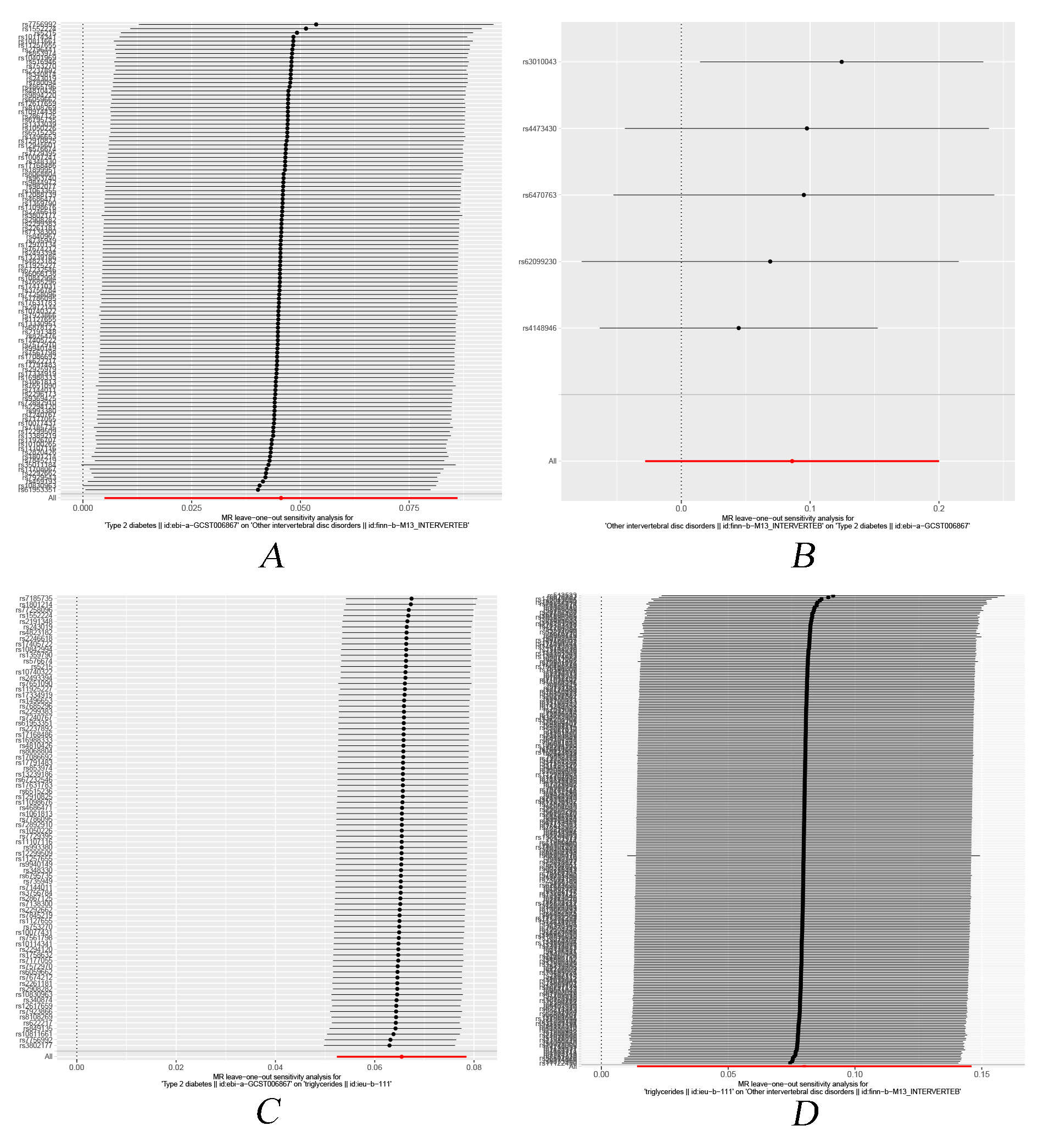


**Fig. S1 Leave-one-out sensitivity analysis forest map.** The leave-one-out method is used to evaluate the excessive impact of a single SNP on MR analysis if the comprehensive effect of the remaining SNPs is consistent with the main effect after removing one SNP. (A) Leave-one-out analysis of T2DM on IVDD. (B) Leave-one-out analysis of IVDD on T2DM; (C) Leave-one-out analysis of T2DM on TGs; (D) Leave-one-out analysis of TGs on IVDD; SNP, single nucleotide polymorphisms; T2DM, Type 2 diabetes mellitus; IVDD, Intervertebral disc degeneration; TGs, triglycerides.
