# Supplementary material for: Genetically predicted triglycerides mediate the relationship between type 2 diabetes Mellitus and intervertebral disc degeneration
Source: Lipids Health Dis. 2023 Nov 14;22:195. doi: 10.1186/s12944-023-01963-4 (PMC10644578; doi:10.1186/s12944-023-01963-4)
Supplement: Supplementary file 9 — Supplementary Material 9 [file 12944_2023_1963_MOESM9_ESM.pdf]

SPRINGER NATURE

Author Services

Editing Certificate

This document certifies that the manuscript

Genetically predicted triglycerides mediate the relationship between type 2 diabetes mellitus and intervertebral disc degeneration

prepared by the authors

Ding-Qiang Chen, Wen-Bin Xu, Xin Chen, Ke-Yi Xiao, Zhi-Qiang Que, Nai-Kun Sun, Jin-Yi Feng, Gang Rui

was edited for proper English language, grammar, punctuation, spelling, and overall style by one or more of the highly qualified native English speaking editors at SNAS.

This certificate was issued on **November 4, 2023** and may be verified on the SNAS website using the verification code **1BE2-C16E-330B-127F-10C2** .

Neither the research content nor the authors' intentions were altered in any way during the editing process. Documents receiving this certification should be English-ready for publication; however, the author has the ability to accept or reject our suggestions and changes. To verify the final SNAS edited version, please visit our verification page at secure.authorservices.springernature.com/certificate/verify.
If you have any questions or concerns about this edited document, please contact SNAS at support@as.springernature.com.

SNAS provides a range of editing, translation, and manuscript services for researchers and publishers around the world.
For more information about our company, services, and partner discounts, please visit authorservices.springernature.com.
